# Modern Text Hiding, Text Steganalysis, and Applications: A Comparative Analysis

**DOI:** 10.3390/e21040355

**Published:** 2019-04-01

**Authors:** Milad Taleby Ahvanooey, Qianmu Li, Jun Hou, Ahmed Raza Rajput, Yini Chen

**Affiliations:** 1 School of Computer Science and Engineering, Nanjing University of Science and Technology, P.O. Box 210094, Nanjing, China; 2 Intelligent Manufacturing Department, Wuyi University, P.O. Box 529020, Jiangmen, China

**Keywords:** modern text hiding, text steganography, text steganalysis, covert communication

## Abstract

Modern text hiding is an intelligent programming technique which embeds a secret message/watermark into a cover text message/file in a hidden way to protect confidential information. Recently, text hiding in the form of watermarking and steganography has found broad applications in, for instance, covert communication, copyright protection, content authentication, etc. In contrast to text hiding, text steganalysis is the process and science of identifying whether a given carrier text file/message has hidden information in it, and, if possible, extracting/detecting the embedded hidden information. This paper presents an overview of state of the art of the text hiding area, and provides a comparative analysis of recent techniques, especially those focused on marking structural characteristics of digital text message/file to hide secret bits. Also, we discuss different types of attacks and their effects to highlight the pros and cons of the recently introduced approaches. Finally, we recommend some directions and guidelines for future works.

## 1. Introduction

**Figure 1:**
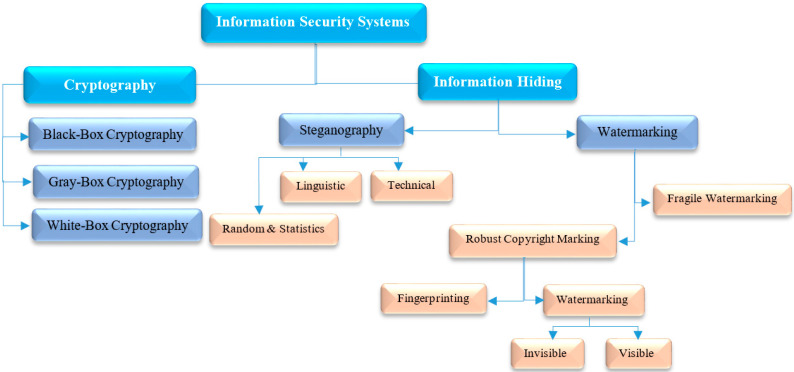
Various categories of information security systems [3,19,20].

Reflecting the new trends and rapid progress in the field of information technology in the form of smart gadgets, communications, and digital content, an extensive environment with the capability to transfer, copy, duplicate, and share information over the Internet has been built, although this revolution in the digital world and the online distribution of digital media also implies that such information is vulnerable to malicious attacks, unauthorized access, forgery, plagiarism, etc. Moreover, digital texts in the form of text messages/files are used in many applications, such as password authentication, chatting, mobile banking, online news, commerce, and so on. However, when we send a text message via short message service (SMS), email, social media, and so on, the information included in the message is transmitted as plain text, exposing it to attacks. In some cases, this information may be sensitive/confidential, such as password authentication, banking credentials, and so on; also, sending such information via SMS or unsecured communication channels is a significant drawback, as neither provides security before transmission. On the other hand, hackers are regularly trying to break the safety of communication channels (e.g., network protocols, SMS, etc.) to access sensitive information during data transmission. Therefore, demand is growing for intelligence and multimedia security studies that involve not only encryption, but also covert communication whose essence lies in concealing data [1,2,3,4,5,6,7,8,9,10,11,12,13,14,15,16,17,18,19]. Recently, information hiding or data hiding in digital texts, known as text hiding, has drawn considerable attention due to its extensive usage, and potential applications in the cybersecurity and network communication industries [20,21,22,23,24,25,26,27,28,29,30,31,32,33,34,35,36,37,38,39,40,41,42,43,44,45,46,47,48,49,50,51,52,53,54,55,56,57,58,59,60,61,62,63,64,65,66,67,68,69,70,71,72,73,74,75,76,77,78,79,80,81,82,83,84,85,86,87,88,89,90,91,92,93,94,95,96,97,98,99,100,101,102,103,104,105,106,107,108,109,110,111,112,113,114,115,116,117,118,119,120,121,122,123,124,125,126,127]. Text hiding is the process of embedding secret data through a cover text or supportable technologies such as network protocols, SMS, etc. so that the existence of the data is invisible/undetectable for adversaries or casual viewers [1,6,8]. It has been widely considered as an attractive technology to improve the use of conventional cryptography algorithms in the area of multimedia security by concealing a secret message/watermark into a cover text file/message to protect confidential information. As depicted in Figure 1, the various information security systems categories that are utilized to protect sensitive data from crackers, deceivers, hackers, and spies are divided into cryptography and information hiding [3]. Cryptography scrambles a plain-text (secret data) into cipher to prevent unauthorized access to its content. On the other hand, information hiding conceals a secret message in a cover medium (e.g., text, image, audio, or video) so that the embedded hidden data trace is unnoticeable/undetectable. Cryptography and information hiding are both similar in the way which is employed to protect confidential/sensitive information. Nonetheless, the invisibility is the difference between both systems, i.e., information hiding involves how to conceal information so it is not noticeable. In practice, information hiding can be classified into watermarking and steganography. The goal of watermarking is providing proof of ownership for the cover media against malicious attacks such as tampering, forgery, and plagiarism (e.g., the embedded watermark indicates the original owner). While, the aim of steganography is the invisible transmission of confidential information so that no one (except an intended recipient) can discover/encode it, i.e., steganography concerns concealing the fact that a medium contains secret data that is invisible/ indiscernible [1,3,41].

During the last two decades, many text hiding algorithms have been introduced in terms of text steganography and text watermarking for covert communication [1,6,8,9,10,11,12,13,14,20,31,36,39,51,91], copyright protection [3,4,5,7,18,20,21,22,23,24,25,26,27,28,29,44,49,50,51,52,53,54,55,56,57,58,59,60,61,62,63,64,65,66,67,68,72,73,74,75,87,88,89,90,91,92,98,99,100,101,102,103,104,105,106,107,108,109], copy control and authentication [31,57,60,74,78,93,94,95,96,97,98].

The main contributions of this paper are summarized as follows:

- We provide a brief review of existing literature on text hiding schema, attacks, text steganalysis, applications, and fundamental criteria.
- We summarize some of the recently proposed text hiding techniques which are focused on altering the structure of the cover text message/file to conceal secret information.
- We present a comparative analysis of the structural based algorithms and evaluate their efficiency with respect to common criteria.

The rest of the paper is organized as follows: Section 2 presents some background literature and related studies on the information hiding area. Section 3 explains various types of text hiding approaches, along with their limitations. In Section 4, we evaluate some of the recently proposed structure-based algorithms and highlight their pros and cons. In Section 5, we give some suggestions for future works. Finally, Section 6 concludes the paper with a summary of contributions.

## 2. Literature Review

In what follows, we present the existing literature on the text hiding area consisting of the schema, fundamental criteria, the Unicode standard, and text steganalysis.

### 2.1. Text Hiding Schema

**Figure 2:**
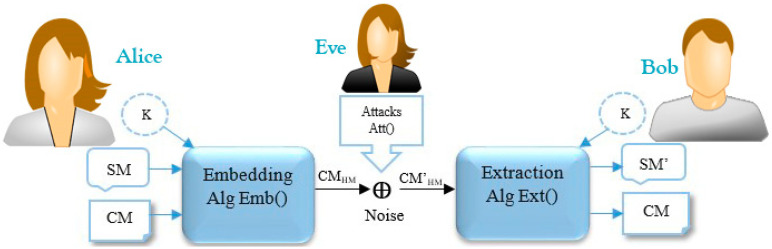
Modern text hiding schema.

The basic scenario of a cryptography covert channel is Simmons’ prisoner problem [108]. Alice and Bob are locked up in two separated cells but are permitted to communicate under the watch of Eve, the prison warden. If Eve discovers the existence of hidden information in a transmitted message, she stops their communication punishes them. Eve is an active warden is she makes noise to make Alice and Bob’s task more difficult. She is a passive warden if she merely detects and investigates the transmitted data [12]. From the digital data hiding point of view, text steganography/watermarking is a different scenario which works based on the practice of hiding a secret message (SM) through a cover message/file (CM) by marking invisible symbols where the trace of embedding the SM is invisible/undetectable by human vision systems. In theory, the Modern Text Hiding schema (*MTH*) can be considered as a form of communication. Figure 2 demonstrates the modern text hiding schema which is represented as *MTHS* [3,9,10,12,47,48,76,77,79].

$$\mathrm{Where},\;MTHS=\langle \{CM,SM,K,CM_{HM}\},\{Att(),CM_{HM},CM\prime_{HM}\},\{Emb(),Ext()\}\rangle$$

As depicted in Figure 2, the modern text hiding scenario consists of two main phases, and a third party phase, namely Embedding “*Emb()*,” Extraction “*Ext()*,” and Attacks “Att().” 

**Algorithm 1:** Pseudocode of Emb()Input: a cover text (*CM*), a secret message (*SM*), a secret key (*K*) Output: a carrier text message or stego-object (*CM_HM_*) which consists of *CM* and *HM* 1. *SM*← Secret Message (e.g., confidential information such as password, banking credentials, etc.); 2. *CM*← Cover Message (e.g., an innocent text message such as prank, joke, etc.); 3. *K*← Secret Key (e.g., a symmetric or asymmetric key algorithm such as One-Time-Pad, AES, DES, etc.); 4. for each $c_{i}\in SM=\left\{c_{1},c_{2},\ldots ,c_{n}\right\}$ do 5. *SM_bits_*← *SM_bits_* + Convert each $SM[c_{i}]$ to a 8-bit string based on the ASCII Code; 6. end for 7. Encrypted *_SM_bits_*← Encrypts the *SM_bits_* based on K using a special encryption function; 8. *HM*← Convert the Encrypted_*SM_bits_* to invisible symbols such as space between words, text color, etc.; 9. *CM_HM_*← Embed the *HM* into the *CM*, where the attacks may not detect/remove it easily; 10. Return CM_HM_;

(1) *Embedding (Emb()):* Alice employs this function to hide an SM into the CM which consists of three stages. In the first stage, the embedding function converts the letters of the *SM* into a binary string (*SM_bits_*). In the second stage, it encodes the *SM_bits_* by using an encryption algorithm based on an optional key(*K*) to secure its content, and produces encoded *SM_bits_*, i.e., One-Time-Pad, AES, DES, etc. Then, it converts the encrypted *SM_bits_* to a hidden message (*HM*) by marking/embedding invisible symbols through the CM. For example, to mark each bit ‘1′, *Emb()* adds two spaces between words and a single space is represented as a bit ‘0′. Finally, it generates a carrier message (*CM_HM_*). Algorithm 1 depicts the sequence of the *Emb()* with more details [1,10,12].

(2) *Attack(Att())*: During the communication process, attackers may attempt to break the security of the *CM _HM_* by decoding or manipulating the *HM* using steganalysis techniques. This process may cause alteration/removal of the *HM* form the *CM’ _HM_*. It is assumed that the attackers do not have any clue about the encoding function, secret key, and *Emb()*. In some cases, attackers employ conventional approaches to guess the invisible/hidden symbols which are statistically distinguishable, and extract/decode the original message, but in practice, this is an impossible task for attackers if the text hiding algorithm utilizes an encryption function during the embedding/extraction process. Algorithm 2 explains the sequence of the *Att()* with more details [1,9,10,12].

**Algorithm 2:** Pseudocode of Att()Input: a carrier message (*CM_HM_*), an estimated secret key (*EK*) Output: a compromised carrier message (*CM’_HM_*), an estimated Secret Message (*ESM*) 1. *HS*← Estimates the hidden/invisible symbols from the *CM_HM_*; 2. for each $c_{i}\in HS=\left\{c_{1},c_{2},\ldots ,c_{n}\right\}$ do 3. *Estimated_SM_bits_* ← *Estimated_SM_bits_ +* Guess the binary string of each symbol based on the $HS[c_{i}]$; 4. *EK_bits_*← *EK_bits_ + Guess* the secret key according to the $HS[c_{i}]$ using the conventional approaches; 5. end for 6. *SM_bits_*← Tries to decrypt the Estimated*_SM_bits_* based on the *ESK*; 7. E*SM*← If it is possible, estimates/decodes the *SM_bits_* using conventional approaches; 8. CM’_HM_← Manipulate the CM’_HM_ in order to remove the HM; 9. Return CM’_HM_, ESM;

3. *Extraction (Ext()):* Bob utilizes this function to extract/discover the original SM from the *CM’_HM._* Since the *CM’_HM_* is transmitted via communication channels, the *HM* may be exposed to attacks, so it is necessary to verify the original *SM* using the same encryption function which already used during the embedding process, i.e., Alice already shared the key with Bob or he has knowledge about the special symbols of the key through the *CM’_HM_*. Two different terms are employed for this function, which are “detection” and “extraction”. However, researchers often define both as similar functions in the literature; we classify them in this way: extraction (*Ext()*) discovers/extracts the *SM* from the *CM’_HM_* and authenticates its integrity, while detection verifies the existence of the *SM* from the *CM’_HM_.* Algorithm 3 outlines the sequence of the *Ext()* with more details [1,9,10,12].

**Algorithm 3:** Pseudocode of Ext()Input: an affected carrier message (*CM’_HM_*), a secret key (*K*) Output: a secret message (*SM’*) 1. HS← Discovers the existing hidden marks/symbols from the *CM’_HM_*; 2. K← Secret Key (e.g., the symmetric or asymmetric key algorithm such as One-Time-Pad, *AES, DES*, etc.); 3. for each $c_{i}\in HS=\left\{c_{1},c_{2},\ldots ,c_{n}\right\}$ do 4. Encrypted*_SM_bits_*← Encrypted_*SM_bits_* + Detects the binary string of each invisible symbol from
$HS[l_{i}]$; 5. K*_bits_*←*K_bits_*+ Utilizes a shared key from Alice or Extracts the secret key from the *CM’_HM_.* 6. end for 7. *SM_bits_*← Decrypts the Encrypted _*SM_bits_* based on *K_bits_* using corresponding decryption function; 8. SM’← Extracts the original *SM* characters from the *SM_bits_* based on their ASCII codes. 9. Return SM’;

### 2.2. Information Theoretic and Modern Text Hiding

This subsection discusses an ideal text hiding system in which the CM and CM_HM_ (cover message with and without the hidden information) are statistically indistinguishable or unnoticeable, i.e., it means that the CM & CM_HM_ have the same probability distribution. We employ the stego-system models presented in [10,127] to clarify this requirement. As depicted in Figure 2, Alice and Bob could exchange messages of a certain kind (called cover message/file) over a public/private channel which is accessible to Eve. Alice wishes to transmit an *SM* in cover of the CM to Bob so that Eve cannot observe whether there exists an *HM* through the CM_HM_. 

The entropy of information theory (*H*) is a popular metric for information measurement introduced by Shannon [128]. It computes the quantity of randomness existing in a message. The equation (1) is commonly utilized to compute Shannon’s entropy [129,130,131]. Let us assume that *CM* consists of unique symbols (or characters) appear into it, i.e., $CM=\left\{c_{1},c_{2},c_{3},\ldots ,c_{n}\right\}$. Herein, $c_{i}$ is the occurrence of $i^{th}$ symbol in all sequences with probability $0<P(c_{i})<1$, $\sum_{i=1}^{n}P(c_{i})=1$, i.e., $P(c_{i})$ is the probability of occurrence for $c_{i}{}^{th}$ element. Thus, the entropy of *CM* can be calculated as follows:

(1) $$H_{CM}=-\sum_{i=1}^{n}P(c_{i})\log_{2}P(c_{i})$$

Let us suppose that Eve does not try to disrupt communication between Alice and Bob, but only attempts to determine if hidden information is being transmitted. In [10], Cachin presented the first formal analysis on the stegosystem in which, depending on the fact that the probability distribution of *CM* and *CM_HM_* is identified, and both cover texts (*CM* and *CM_HM_*) are statistically close. Later in [127], Ryabko and Ryabko commented that the *CM* and *CM_HM_* are statistically indistinguishable. They assumed that Alice has access to an oracle which makes independent and identically distributed cover texts (*CM* and *CM_HM_*) based on some fixed but unknown distribution μ. The *CM/CM_HM_* consists of some symbols that belong to some (possibly infinite) alphabet A. Alice wishes to employ this source as cover to transmit hidden messages. An *HM* is a sequence of symbols or letters from B = {0,1} produced independently by equal probabilities of ‘0′ and ‘1′. Also, it is assumed that Alice encrypts *SMs* using a key shared only with Bob, i.e., similar to a common cryptosystem scenario. If Alice utilizes the Vernam cipher then, the encrypted *SMs* are certainly produced according to the Bernoulli (1/2) distribution, while if Alice employs “modern block” or “stream” ciphers, the encoded sequence thus “looks like” a sequence of random Bernoulli (1/2) trials. Herein, “look like” means that it is indistinguishable in polynomial time, or that the resemblance is proved experimentally by statistical data, known for all broadly utilized ciphers [132,133]. Eve or a third party is monitoring all messages transmitted from Alice to Bob and is attempting to detect whether *SMs* are being passed in the *CM* or not. In the best case scenario, if the text hiding technique does not change the *CM_HM_* by embedding the *SM* it means that the *CM* and *CM_HM_* have the same probability distribution (*μ*), hence, it is impossible to distinguish the presence of the *HM* from the *CM_HM_*. In [127], the authors confirmed that if the alphabet A is finite, then the average number of invisible/hidden symbols per character *L_n_* goes to Shannon’s entropy *H*(*μ*) for the source μ, as n goes to infinity; as a result of this statement the definition can be expressed as follows: $H(\mu )=-\sum_{a\in A}\mu (a)\log_{2}\mu (a).$ Since, some existing text hiding techniques embed invisible symbols into the CM for marking the *SM_bits_*, the trace of embedding into *CM_HM_* is visually imperceptible, but, in practice, the *CM* and *CM_HM_* are statistically distinguishable, and their variation rate can be calculated by Equation (2), i.e., a Jaro similarity function [29,125,126].

### 2.3. The Unicode Standard

Unicode is a universal standard which has been introduced for the processing, encoding, and handling of the digital texts expressed in most of the world’s writing systems from 1987 until now [100,101,102,103,104]. In other words, the Unicode standard is an encoding system which designed to support the worldwide display, processing, and interchange of the texts with different languages and technical disciplines. Moreover, it also supports classical and historical characters of many languages. Necessarily, Unicode is required by the various Internet protocols (e.g., TCP/IP, SMTP, FTP, and HTTP, etc.) and implemented in all operating systems (e.g., Android, Windows, iOS, and BlackBerry) and programming languages for processing and displaying digital texts. This standard consists of three different encoding forms, UTF-8, UTF-16, and UTF-32, for which Unicode provides 17 planes, each with “65,536” possible letters (or ‘code points’). Therefore, it affords a total of 1,114,112 possible symbols/characters in various formats such as numbers, letters, emoticons, and a vast number of current characters in different languages, i.e., the UTF-8 presents one byte for any ASCII character, which have the same code values in both ASCII and UTF-8, and up to four bytes for other symbols [1,2,3,4,5,6,7]. In the Unicode, there are special zero-width characters (ZWC) which are employed to provide specific entities such as Zero Width Joiner (ZWJ), e.g., ZWJ joins two supportable characters together in particular languages, POP directional, and Zero Width Non-Joiner (ZWNJ), etc. Practically, the ZWC characters do not have traces, widths or written symbol in digital texts [1,2,3,4,5,6,7,8,11,15,18,25,26,27,28,33,34,41,42,43,50,51,52,53,54,55,56,57,58,59,60,61,62,63,64,65,66,67,68,86,87,88,89,90,91,92,93,94,95,96,97,98,99,100]. Recently, many text hiding techniques that utilize social media, email, SMS, as communication channels have been introduced [1,6,8,11,20,36,37]. In a particular social media platform, if it employs the Unicode standard to process digital texts in different languages, then the ZWCs represent invisible written symbols. Otherwise, they might just show some unusual symbols. As listed in Table 1, We have collected all of the utilized characters from the literature and tested them by Java programming in .txt, MS .docx, and HTML files, i.e., the ZWCs have no trace with respect to the written symbol. In practice, when ZWCs/special spaces are employed for embedding a secret data in the cover text, the default encoding used must one of the Unicode encodings like UTF-8, UTF-16, or UTF-32. In case of attack, if a malicious user copies a target text which contained some ZWCs in the new host file, then these characters will be considered as the Unicode encoding and show an invisible text trace. Otherwise, they display some unsupported characters and raise suspicions about the existence of secret information [1,3,6,7]. 

Based on our experiments, Gmail blocked the “U+200B” character, and the Apple iOS does not allow one to transmit the “U+200D” character. Moreover, we highlighted the special Unicode spaces between double quotation marks and changed the font color to show their width, but they are transparent in practice.

These days, social media play a vital role in the new digital world; the end users are using it to keep in touch with their friends or make some new friends. Sometime to exhibit confidence they post/share their latest accomplishments with friends. Everyone utilizes it differently. Some end users are employing social media as per their priorities and awareness to achieve their means. Further, these tools are all handy for online advertisements, payments, and business systems. At the early stages, social media was not that big yet, but now people can use it for almost anything in their daily life. Also, people’s cultures have been more impacted than anything else by social media in recent years. Large media companies are not expected to go away overnight, nor will the demand to communicate by smartphone or meet people in person, but social media provides one more means of engaging with users on this enormous planet, and if employed effectively could give all a more desirable option in how to live and communicate to each other in the digital world. Since the text message in the form of SMS, chat, email, and so on, has become a popular and easy form of communication, concerns about data leakage attacks, such as hacking, hijacking, and phishing, have emerged [1,6,8,11]. Table 2 lists the text character limitation of social media and messenger apps which support the Unicode standard to process digital texts in different languages (except for ‘Twitter’ and ‘Telegram’).

### 2.4. Text Hiding Applications 

Text Steganography algorithms are applicable in many applications. The following points are the most significant applications of text steganography.

#### 2.4.1. Hidden Communication

Text hiding could be utilized to communicate hidden information over public networks such as the Internet. One may embed secret bits into an unnoticeable text message/file which is routinely transmitted over such networks: a greeting, joke, story, etc. Since the text messages/files are sent using unsecured communication channels such as SMS, social media and so on, they are exposed to attacks. Users of such techniques may consist of intelligence or people who are subject to censorship such as detectives, journalists, judges, and so on [1,6,10,11,12].

#### 2.4.2. Network Covert Channels

Text hiding can be used to make covert channels that provide unexpected stealthy communication over the networks. Recently, covert channels were employed by cyber-attacks, i.e., to permit a covert transmission of malware data. Nevertheless, they could also be applied for legitimate goals, such as transmitting illicit information under Internet censorship [14,98,107].

#### 2.4.3. Unauthorized Access Detection

Text hiding could also be employed to detect unauthorized access to sensitive documents over private networks. For example, sensitive/confidential documents in a governmental or commercial organization can be marked with identifiers that are difficult to detect. The aim is to trace unauthorized access/use of a sensitive document to a specific user who may have obtained a copy of the marked document. The receiver of such documents should not be aware of the existence of the identifiers [12,40,64].

### 2.5. Text Hiding Criteria 

**Figure 3:**
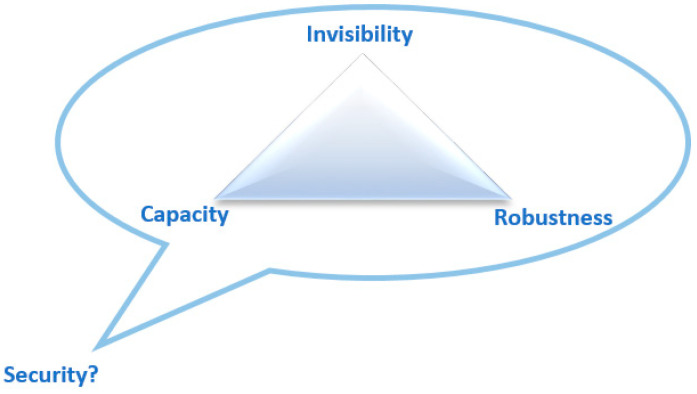
Evaluation criteria of text hiding algorithms.

There are many things to be considered when programmers design a text hiding algorithm. However, the fundamental criteria can be easily found in recently introduced algorithms: invisibility, embedding capacity, robustness, and security [1]. The communication channel over which the *CM_HM_* is transmitted can be noisy or noiseless, for the case of an active or a passive warden, respectively. Also, the steganographer capability to select the CM is often restricted if not altogether non-existent [12]. In a network (private or public) application, the CM is produced by a steganographer (in a public channel) or a content provider (in a private channel), i.e., for the private network application, the authority responsible for document security. Moreover, for the covert channel application, the *CM* is created by the computer, not by the infringer. Depending on these applications, a trade-off must be sought for satisfying the criteria on any point inside the magic triangle as depicted in Figure 3 [1,7,10,12].

#### 2.5.1. Invisibility

Quantifying an attacker or Eve’s capability to discover/detect the existence of *HM* is called invisibility (or imperceptibility/detectability/transparency), i.e., the embedding trace of an *HM* in the *CM_HM_* must be invisible and avoid raising the suspicions of human vision systems. In other words, invisibility refers to how many perceptual modifications are made in the *CM_HM_* after embedding an *HM*. Practically, it cannot be measured numerically. The best way of analyzing the degree of invisibility is to compare the variation of *CM* and *CM_HM_*, i.e., with and without the *HM* [1,7,10,12]. In some literature, researchers utilized the Jaro–Winkler Distance (or Jaro Similarity) for analyzing the similarity of the original CM and CM_HM_. It can be defined as follows:

The Jaro distance *d_j_* of two given strings $s_{1}=Lenngth(CM)$ and $s_{2}=length(CM_{HM})$ is:

(2) $$d_{j}=\{\begin{array}{c} 0\;if\;m=0\\ \frac{1}{3}\left(\frac{m}{\left|s_{1}\right|}+\frac{m}{\left|s_{2}\right|}+\frac{m-t}{m}\right)\;else \end{array}$$

where, m is the number of matching characters, and *t* is half the number of transpositions. Two letters from *CM* and *CM_HM_*, respectively, are considered identical only if they are equal and not higher than $\left\lfloor \frac{\max\left(\left|s_{1}|,|s_{2}\right|\right)}{2}\right\rfloor -1$. Each letter of *CM* is compared with all the matching characters in *CM_HM_*. The number of identical letters (but in different sequence order) divided by 2 specifies the number of transpositions. If the $d_{j}$ is “0”, then the *CM* and *CM_HM_* are not similar, and “1” means both are exactly the same. A *d_j_* nearest to 1 represents that the *CM* and *CM_HM_* are closely similar [29,125,126]. However, it does not consider the similarity of the structural techniques due to the fact they do not modify the characters of the *CM* to hide the *SM_bits_*.

#### 2.5.2. Embedding Capacity (EC) 

The number of secret bits which can be embedded in the *CM* is called embedding capacity (or payload). This feature could be measured numerically in units of bit-per-locations (BPL) or character-per-locations (CPL). Location means a changeable feature (or character/word) which can be considered as an embeddable location (*EL*) in the *CM* such as between words, after special characters, etc. Nevertheless, a text steganography algorithm provides a large *EC*; it is not efficient if it modifies the *CM* profoundly [1,7,10,12]:

(3) $$EC_{CM}=BPL\times EL_{CM}\;\mathrm{or}\;EC_{CM}=CPL\times EL_{CM}$$

#### 2.5.3. Distortion Robustness (DR)

Multiple attacks may occur on the *CM_HM_* while it is transmitted on the channels where it may be exposed to a hazard that could destroy the *HM*. Moreover, attackers may try to manipulate the *HM* rather than remove it. Therefore, any type of distortion might occur deliberately or even unintentionally on the *CM_HM_*. A robust text hiding algorithm makes the *HM* extremely difficult to alter or destroy. It could also be measured numerically based on losing or removing probability P(L). In other words, P(L) is the probability of how much proportion of the hidden symbols has been lost from *CM_HM_*. Let us suppose that the number of *ELs* in the *CM* is *NL*, the length of the *CM* is stand as TC. Thus, the P(L)=NL/TC and the P(DR) can be computed as follows [1,3]:

(4) $$P(DR_{HM})=[1-P(L)];\;1<NL<TC,NL\in \mathbb{N},TC\in \mathbb{N}.$$

#### 2.5.4. Security

There is a certain level of safety that prevents attackers from detecting the *HM* visually or from removing it from the *CM_HM_* (i.e., quantifying decoding reliability in the presence of channel noise when Eve is an active warden). This measure depends on three other criteria: invisibility, embedding capacity, and distortion robustness. An efficient steganography algorithm must provide an optimum trade-off among these criteria. If a method affords a large EC, the embedding trace of *HM* is invisible, and robustness is high, then the security of the algorithm can be calculated using Equation (4). In modern text hiding techniques, a cryptosystem can be utilized to protect secret bits against decoding attacks. In practice, the encryption function is employed to secure the *SM_bits_* before embedding them into the *CM*, and alters the sequence of the secret bits such that they can only be extracted by the corresponding decryption function [1,12]. Decoding Probability (DP) is the probability of decoding an original *SM_bits_* by guessing attacks. Let us suppose that, an attacker speculates a message may contain an *HM* (e.g., he/she does not have any clue about the approach that was utilized to conceal the SM). Moreover, the attacker may try to decode the *SM* using conventional approaches or guessing the *SM_bits_* (using probability distribution analysis) from the invisible symbols or features. Since an encryption function is used to secure the *SM_bits_* based on a secret key (K), it is impossible to decode the original SM from the encrypted *SM_bits_* without having the secret key and the corresponding decryption function. If NS is the length of the SM binary, the P(*DP*) for guessing a correct encrypted binary string of the SM can be calculated as follows:

(5) $$P(DP)=\sum_{i=i|k}^{NS}(\frac{1}{2^{i}}{)}^{\frac{NS}{i}},i:\exists_{k\in \mathbb{N}|i\times k=NS},i\in [1,\ldots ,NS],i\in \mathbb{N}$$

#### 2.5.5. Computational Complexity

The computational cost or complexity is the least significant measure for the next-generation smart devices such as computers, smartphones, tablets, etc. Nevertheless, there could be many pages in some text files; thus, it is preferable that steganography/watermarking techniques be computationally less complex. It is obvious that the long text files need more hardware or software resources, that is, they have higher computational complexity. Generally, the less complex approaches are employed for resource-limited systems such as embedded microprocessors and mobile devices. Let us assume that the NS is the length of the SM, and the LC is the length of CM; Then, the minimum computational cost for the Emb()/Ext() is *O*(*NS*×*LC*) due to need for searching *LC* times to finding the embeddable locations for marking each letter of the *SM* (or *SM_bits_*). However, the complexity of the additional costs such as encryption function, the dictionary of words, etc. must be considered in those techniques utilizing them during the embedding/extraction process [3,46,49].

### 2.6. Modern Text Hiding & Kerckhoffs’s Principle

Since modern steganography/watermarking is a key-based algorithm similar to cryptography, the question for adhering to Kerckhoffs’s principle may emerge [1,17]. Kerckhoffs introduced for the first time the prudent tradition known as “Kerckhoffs’s principle” for cryptology in which an ideal crypto-system should be secure even if everything about the system is identified to the public except the secret key [104]. Therefore, an ideal text hiding algorithm should guarantee that it adheres to Kerckhoffs’s principle. Even if the attacker identifies how the stego-system works, it should not be possible to discover the system design. As depicted in Figure 2, the *CM_HM_* is just like *CM* and the original *CM* is not sent to the recipient in the transmission process—thus any receiver cannot compare the *CM_HM_* with the original *CM*. Therefore, the original *SM* is only extractable by the key which is encrypted using a specific algorithm, so without knowing the original secret key, no one could break a modern text hiding algorithm [10,12,17,104].

### 2.7. Text Steganalysis and Attacks

In contrast to text steganography (or watermarking), text steganalysis is the estimation process and science of identifying whether a given text message/file has hidden information in it, and, if possible, extracting/recovering the secret message. This term is similar to the way cryptanalysis is utilized in cryptography. In practice, the text steganalysis is a complicated task, because of the wide variety of digital text characteristics, the extensive variation of embedding approaches and usually, the low embedding distortion. In some cases, text steganalysis is possible due to the fact data embedding modifies the statistics of the cover message/file. In other words, the existence of embedded symbols (e.g., those techniques which modify the *CM* in order to hide the secret bits) still makes an original *CM* and its corresponding *CM_HM_* different in some aspects, though this is often imperceptible to the human vision system. Concerning the application, steganalysis methods could be typically classified into two categories: specific and universal. While the former attempt to break a unique watermarking/steganography algorithm, the latter aim to thwart all watermarking/steganographic algorithms. In practice, specific techniques achieve higher detection accuracy as compared to universal ones due to the fact they use prior knowledge of how the particular target algorithm works. However, the universal steganalysis is more attractive in practical application since they could operate independently of the embedding method and even be generalized to unknown steganography/watermarking approaches [16,17,105,106]. From a steganalysis point of view, we can classify the possible attacks into three types, including visual attacks, structural attacks and statistical/probabilistic attacks.

#### 2.7.1. Visual Attacks

The visual attacks or Manipulation by Readers (MBR) refers to a human factor, often a viewer who could perceptually (visually) observe the modifications through the *CM_HM_* or stego object. These modifications may consist of syntactic, semantic paraphrasing, lexical, rhetorical changes, and so on. Let us assume that an attacker has complete access to the *CM_HM_*, and if he suspects that there exist some unconventional modifications through the *CM_HM_*, then, he might manipulate it (i.e., it could be an intentional deletion, insertion, or re-ordering of words/characters). In practice, any types of manipulations through the *CM_HM_* may destroy the *HM* [1,3,17,23,111].

#### 2.7.2. Structural Attacks

This attack involves modifying the layout of the *CM_HM_*. In some cases, attackers may change the formatting (e.g., font or copy from the *CM_HM_* to a new host file), encoding (e.g., ASCII, UTF-8, UTF-16, etc.) of the *CM_HM_* that may lead to destroying the *HM* [1,3,17].

#### 2.7.3. Statistical Attacks

This attack works based on the possibilities of guessing a correct *SM* in which the adversary can discover occult symbols from the *CM_HM_* by considering the number of words, spaces, and so on. Basically, this attack utilizes the knowledge of existing approaches to decode/guess the original *SM* using probability distribution functions [10]. When the *CM_HM_* does not show any visible alterations, the adversary processes the characters/letters of the *CM_HM_* to analyze the statistical variations, i.e., it may happen during the data transmission using *MITM* attacks [1,31,110]. Let us suppose that a *CM*_HM_ contains *NC* characters, *NH* hidden symbols (spaces, zero-width characters, etc.). If the length of the *SM* is *NS*, then there are 2^NS^ possible secret messages which can occur. Thus, the number of possible solutions (*NP*) for guessing the *SM* can be obtained as follows:

(6) $$NP=k\times 2^{NS},SM=\{c_{1},c_{2},\ldots ,c_{NS}\}.$$

Moreover, the number of guessing the *NH* symbols from the *CM*_HM_ can be computed using Equation (7):

(7) $$P(NH,NC)=\left(\begin{array}{c} NC\\ NH \end{array}\right)=\frac{NC!}{(NC-NH)!\times NH!},NH\leq NC.$$

Therefore, the probability of guessing a correct SM (i.e., cracking probability) from the *CM*_HM_ can be calculated as follows:

(8) $$P(SM)=\frac{1}{NP}\times \frac{1}{P(NH,NC)}=\frac{1}{2^{NS}\times \frac{NC!}{(NC-NH)!\times NH!}}.$$

If a text hiding algorithm utilizes an encryption function to secure the *SM_bits_* using a secret key, then the *P*(*SM*) is equal to zero (i.e., it is impossible to break) [10].

## 3. Various Types of Text Hiding Techniques

**Figure 4:**
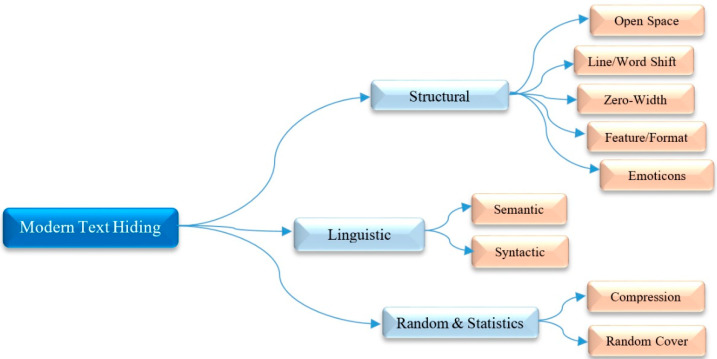
Various types of text hiding techniques.

Technically, there are various algorithms employed for information hiding in the form of the text steganography and text watermarking in the literature [3,19,46,49]. In practice, these two terms are different in the goal of embedding hidden data into a cover text message/file, where the concern is the protection of cover text content (called “text watermarking),” and the concern is the hidden transmission of the secret information (called “text steganography”). We can classify the existing text hiding techniques into one of the categories in Figure 4, namely, structural, linguistic, and random and statistics [2,3,20,29,49].

### 3.1. Structural Techniques

Structural or format-based algorithms involve modifying the layout features or format of the *CM* to mark/hide the *SM_bit_*_s_, i.e., based on the Unicode or the ASCII encoding without altering the sentences or words. These features consist of word spacing, line spacing, font style, text color, and so on [1,2,3,4,5,6,7,8,11,20,34,41,54,65,66,100,112,113,114]. Herein, we classify the structural-based techniques into four categories, including, open space, line/word shift, zero-width, feature/format, and emoticons.

#### 3.1.1. Open Space

The open space (or white space)-based techniques utilize special Unicode spaces to mark/embed secret bits into the *CM*, i.e., for example: between words, end of the sentences, and so on. Many approaches have been introduced using the idea of open space during the last two decades. In practice, these techniques provide high invisibility, low embedding capacity and modest robustness against visual attacks. Moreover, they can be applied in multilingual digital texts [6,7,15,27,34,41,54,65,66,100].

#### 3.1.2. Line/Word Shift

Line/Word shift-based techniques involve shifting lines vertically or words horizontally to hide the *SM_bits_* through the cover text file. In other words, these techniques evaluate the scanned images of the printed documents to extract or reveal the watermark. In practice, they are not applicable in digital texts because if someone copies the carrier text to a new host file, the extraction algorithm cannot discover the hidden information. From the criteria point of view, these techniques typically provide low embedding capacity, high invisibility, and low robustness against structural attacks [112,113,114].

#### 3.1.3. Zero-Width

The zero-width-based techniques employ the ZWC Unicode characters to embed/mark the *SM_bits_* into the cover text. From the text processing point of view, the ZWCs have no text trace (written symbols) and can be embedded in different locations through the *CM*, but, they can be processed by programming analysis of the *CM_HM_*. These approaches can be utilized in multilingual texts and various text processing platforms such as social media, email, SMS, etc. For example, a zero-width steganography technique called AITSteg was proposed in [1], which utilizes the ZWCs to embed a long *SM_bits_* in front of a short *CM*. Since the ZWCs have invisible text traces through the *CM*, they can be embedded using the max number of letters in the channel (e.g., SMS, Facebook, etc.). In practice, the zero-width-based approaches provide high invisibility, high embedding capacity and higher robustness against structural attacks [1,4,25,26,27,28,33,55,56,91,115].

#### 3.1.4. Feature or Format

The feature/format-based methods involve modifying some features of the cover text such as font size, style, color, etc. that could be altered to conceal secret bites [18,21,24]. For instance, the dotting feature of the Arabic texts can be used for marking the *SM_bits_* by displacing letter points and diacritics [116,117,118,119]. Since the structure of the Arabic language is similar to the Persian and Urdu languages, these languages use the same point letters. Several techniques have utilized point letters to mark/embed secret bits by displacing the position of a point a little bit vertically high concerning the standard point position through the *CM* [15,88,90,92]. In practice, these techniques provide high invisibility (except for color-based ones), higher embedding capacity, and low distortion robustness against structural attacks. Color-based algorithms are also vulnerable to visual attacks [111].

#### 3.1.5. Emoticons or Emoji

Emoticon or emoji-based approaches utilize the emoji symbols to embed the *SM_bits_* through the *CM*. These days, end users employ emoticons or emoji symbols in daily conversations instead of typing their feelings. Recently, several algorithms have been introduced using the cover of emoticons to mark secret bits through the *CM*. For instance, the techniques presented in [8,120,121,122] generate a random text consisting some words as a *CM*, and also, they convert the letters of the *SM* into emoticons based on a predefined pattern (e.g., A = “
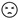
”, B = “
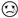
”, C = “
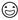
”, and so on.). Moreover, they embed the produced emoticons between words through the *CM*. Although these approaches have high embedding capacity, they suffer from visible transparency (low invisibility), and low distortion robustness against visual attacks.

### 3.2. LinguisticTechniques

Llinguistic or natural language processing-based algorithms alter the syntax and semantics characteristics of the text content. The text typically consists of several words, sentences, verbs, nouns, adverbs, adjectives, and so on. Several linguistic-based approaches have used characteristics such as synonyms, abbreviations, the similarity of words, and so on, to embed secret bits into a *CM* [17,62,70,71,80,81,82,83,84,85,106,109]. In general, we can classify the linguistic based approaches into two types: syntactic and semantic.

#### 3.2.1. Semantic

Semantic methods work based on the specific language characteristics by modifying the semantic attributes of the *CM* to mark/embed the *SM_bits_*. These attributes include the spelling of words, abbreviations, synonyms, acronyms, and so on [62,70,71,75,82,84]. The advantage of the semantic-based methods is that they protect the *HM* against retyping attacks or the use of OCR software [111]. Moreover, these methods provide low embedding capacity, high invisibility and high robustness against structural attacks, but they modify the original meaning of the *CM*.

#### 3.2.2. Syntactic

Syntactic approaches involve modifying the *CM* without significantly changing the meaning or tone of the text content. In different languages, there are some syntactical compositions in their text structures, which are specified by the language and its specific conventions [3,20,81,82,83]. For instance, a method presented in [123], which utilizes the similarity of La word in the Arabic/Persian text. In this approach, the primary form of “La” (“لـا”) is employed for hiding a bit “0,” and specific form of the word “La” (“لا”) is employed for concealing a bit “1” through the *CM*. In practice, the syntactic-based techniques have low embedding capacity, high invisibility and high robustness against structural attacks. They are also vulnerable to visual attacks.

### 3.3. Random and Statistics Techniques

The random and statistics generation algorithms employ the statistical features of the *SM* to generate the *CM* automatically. In other words, these techniques do not require an existing *CM*, and utilize the structures and properties of a particular language i.e., what is the past format of a verb, how to generate the sentences, etc. [21,23,24,29,34,35,39,47,51,124]. In general, these methods have higher computational complexity which consumes more time and space to generate a *CM*. 

#### 3.3.1. Compression 

The compression-based methods utilize a lossless compression algorithm such as Huffman coding, Lempel–Ziv–Welch (LZW), arithmetic coding, etc. to hide the *SM_bits_* into the *CM* [21,24,34,35,39]. For example, a LZW compression-based steganography algorithm presented in [39] embeds the *SM_bits_* in e-mail addresses. This method considers the statistical distance for each letter of the *SM* such that a dependent ‘distance’ of the same letter in the cover text is computed. Therefore, a ‘distance vector’ is derived for the *SM* and a ‘distance matrix’ is produced for each CM. A text which gives the highest frequency of the distance values is finally selected from the text-based as a *CM* as well as the stego key. Moreover, the LZW code is computed for this distance matrix and the produced bits are divided into blocks of 12 bits including 9-bit, and 3-bit segregations. These segregations are employed to choose the domain name and the user-name from the available options to make a valid e-mail address. In practice, the compression-based algorithms require high computational complexity, and they are not efficient for hiding the *SM* in short cover texts. However, they provide high invisibility, optimum capacity, and low robustness against structural attacks.

#### 3.3.2. Random Cover

The random cover-based techniques work by generating a cover according to the *SM* letters. Initially, the *Emb()* must generate a *CM* based on the *SM* letters, and then embed/mark the *SM_bits_* inside the *CM* [23,47,51,124]. For instance, a random cover generation technique called AH4S introduced in [51], which employs the structure of the omega network to conceal the *SM_bits_* in a generated *CM*. This method picks a character from the *SM* and utilizes the omega network to generate two related letters based on a picked character. Moreover, it searches in a predefined dictionary for an appropriate English cover word to hide the two generated characters and reproduces the same process for all characters of the *SM*. This approach generates a long unknown text for a short *SM* and increases suspicions for readers/attackers. Practically, the random cover-based techniques provide perceptual transparency (low invisibility), low capacity, and high robustness. Moreover, they have high computational complexity for generating the *CM* during the embedding/extraction process.

### 3.4. An Empirical Comparison

**Figure 5:**
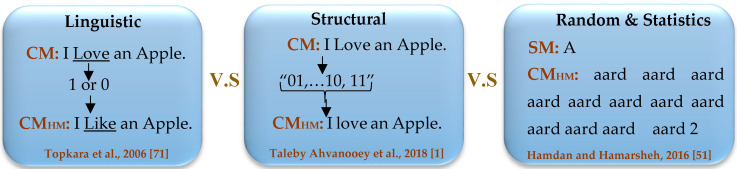
An empirical comparison between linguistic, structural, and random & statistics algorithms.

To demonstrate the variations between various types of text hiding techniques, we summarized an example of embedding method for each category as depicted in Figure 5. Let us assume that the *Emb()* of each approach hides an *SM* (or *SM_bits_*) through the *CM*, and each one produced a *CM_HM_*, which are different from the other ones. Thus, we can observe that there are some pros & cons for each category as listed in Table 3. We rated each type empirically based on the criteria, including, invisibility (Imperceptible, Perceptible), EC (Low, Modest, and High), and DR (Low, Medium, and High).

As listed in Table 4, we summarized some highlights and limitations for each category separately by considering their characteristics and their applications. 

## 4. Efficiency Analysis of Recent Structural Techniques

During the last decade, many structural based text hiding algorithms have been introduced, and a few methods proposed in the linguistic-based and random and statistics-based categories. There are some reasons for that: some limitations such as low EC, altering the meaning of the *CM*, generating an unknown *CM*, etc. which make them inefficient for some applications might be the main reason. The second reason is that they both work based on the features of the language of the *CM/SM* to hide the *SM* that require some additional needs such as a predefined dictionary, dataset, etc. In what follows, we summarized the recent structural-based techniques that can be applied in multilingual texts and various applications.

Por et al. [7] proposed a text-based data hiding technique called UniSpaCh, which generates a binary string of the *SM* and isolates it by 2-bit classification (i.e., “10, 01, 00, and 11”). Moreover, it substitutes each 2-bit with a special space (e.g., Thin, Hair, Six-Per-Em, and Punctuation). Finally, it embeds the additional spaces into predefined locations such as inter-words, inter-sentences, end-of-line, and inter-paragraphs into the MS Word file. However, this technique gives high invisibility, high robustness against structural and visual attacks, but it has low EC rate (two bits per spaces) and is not applicable to embed a long *SM_bits_* into a short *CM*. 

Odeh et al. [33] suggested a novel text steganography algorithm called ZW_4B using the ZWCs characters that hides *SM_bits_* inside an MS Word file. As depicted in Table 5, this algorithm employs four ZWCs to mark four bits of the SM_bits_ between letters in the *CM* file. For instance, the algorithm inserts all the four ZWCs after a letter through the *CM*, then it represents the hidden code is “0001”, if it embeds three ZWCs, then it marks “0001”, and so on. In practice, this technique provides high invisibility, higher embedding capacity, and can be applied in multilingual texts. However, it suffers from low robustness since only the embeddable location is between letters. Moreover, this method can preserve the embedded bits against structural attacks.

Naqvi et al. [29] presented a multi-layer text steganography scheme called MHST using homomorphic encryption, which replaces the characters of the *SM* with the letters of the *CM* to hide it. In the experimental results, the authors claimed that this algorithm provides high embedding capacity, imperceptible transparency, and high robustness against structural attacks, but it suffers from visual or MBR attacks. i.e., if an attacker manipulates a portion of the *CM_HM_*, the extraction process of the *SM* might fail due to possibility of removing some characters of the *SM* through the *CM*.

Odeh and Elleithy [90] introduced a text steganography method called ZWBSP that embeds the *SM_bits_* by adding a ZWC (U+200B) beside of the normal space (U+0020) between words through the MS Word file. This algorithm considers the embeddable location before/after the standard space between words based on a predefined pattern as outlined in Table 6. In practice, this method gives high invisibility, low EC, and medium robustness. Moreover, it is applicable in different languages, and protects the embedded *SM_bits_* against structural, and visual attacks.

Rizzo et al. [5] provided a text watermarking approach called TWSM which can embed a password based watermark in a Latin-based *CM*. This approach utilizes the homoglyph Unicode characters and special spaces for marking the watermark/*SM_bits_* in the *CM*. The researchers claimed that this approach could conceal a watermark (64 bit) into a short *CM* with only 46 letters and, also, it provides high invisibility and high capacity. However, it is vulnerable to structural attacks (e.g., modifying the font type of the *CM_HM_* causes the *SM_bits_* to be lost), and visual attacks. Due to its use of homoglyph characters, this method could only be applied in Latin-based cover texts. Later on, Rizzo et al. [6] used the same algorithm [5] to mark/embed a watermark in social media platforms.

In [58], Alotaibi and Elrefaei proposed two watermarking techniques based on modifying the cover text using ZWCs and Unicode spaces. In the first algorithm, the dotting attribute of the Arabic language applied in [15] is utilized to enhance the capacity of the previous work. Moreover, the ZWNJ is employed to mark/embed before and after the normal space depending on the letter which is pointed or unpointed. In the second algorithm, four Unicode characters are utilized to add next to normal space (e.g., ZWNJ, Thin, Hair, and ZW), herein is called 4-SpaCh. Every four bits from the *SM_bits_* are marked/embedded by corresponding the Unicode characters and order: the 1st bit is denoted by the ZWNJ, the 2nd bit by Thin space, the 3rd bit by Hair space, and the 4th bit by ZW space. Hence, if the algorithm embeds all four spaces, then it represents a ‘1′, otherwise a ‘0′. In practice, the second algorithm can be utilized for embedding in multilingual texts due to employing the Unicode characters to mark the *SM_bit_*_s_ into the *CM_HM_*. This technique has higher EC, high imperceptibility, and low DR against visual attacks, i.e., if an attacker manipulates a portion of the *CM_HM_* (consisting of some spaces), then it causes extraction by the corresponding *Ext()* to fail for the whole of the *SM*. 

Shu et al. [11] presented a text steganography algorithm by employing a combination of white-space and extended-line called WS_EL which provides secure communication on social media [23]. This approach generates a binary *SM* string, and embeds an additional white space between words, at the end of a line, and at the end of the paragraph to mark the *SM_b_*_its_. In the experimental results, they claimed that this approach gives optimum EC, high invisibility, but, it also has low DR against visual attacks.

Taleby Ahvanooey et al. [1] proposed an innovative text steganography algorithm called AITSteg which can hide a long *SM* through a short *CM* for sending via social media. This method generates an *SM* binary string by the “Gödel” function and encodes the *SM_bits_* by a dynamic random key generation algorithm. Also, it converts the encoded *SM_bits_* to ZWCs based on a predefined pattern as outlined in Table 7, and embeds them in front of the *CM*. In this work, the authors evaluated the AITSteg on fifteen social media (or messenger apps), and pointed out that only two social media including Twitter and Telegram do not support the employed ZWCs. From the experimental results, it can be concluded that the AITSteg provides high invisibility, high EC, and high DR against visual and structural attacks.

Kumar et al. [34] suggested a text steganography scheme called 4&3SpaCh which extended the UniSpaCh [7] by efficiently employing the Unicode characters. This scheme conceals the *SM_bits_* into the MS Word file by considering the embeddable locations, including, inter-sentence, inter-word, end-of-line, and inter-paragraph spaces. As listed in Table 8 and Table 9, the authors utilized two different patterns to mark the *SM_bits_* through the *CM*. However, this scheme provides high imperceptibility, and higher EC compared to the UniSpaCh, and high DR against structural attacks. However, it generates some unconventional gaps between words through the *CM_HM_*, which causes increased visual attacks.

Patiburn et al. in [13] developed an emoticons-based text steganography scheme called EM_ST which generates a random text consisting of some words as a *CM*. Moreover, it converts all the *SM* characters into emoticons based on a particular pattern (e.g., A=“
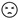
”, B=“
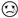
”, C=“
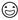
”, and so on.) and, thus embeds the emoticons between words through the *CM*. Practically, this scheme presents high EC, and visible transparency (low invisibility), and it suffers from low DR against visual attacks.

To demonstrate the embedding trace and invisibility of the explained algorithms, we implemented them on some cover text examples. Herein, the implementation means the evaluation of selected algorithms based on their corresponding *Emb()/Ext()* approaches. 

To ensure a fair comparison between existing structural algorithms, we considered those which could be applied in multilingual cover texts. Let us suppose that we wish to hide as *SM_bits_* = Ab = ”01000010 + 01100010”, then after implementing the aforementioned approaches on highlight cover text examples, the embedding trace of each method highlighted as depicted in Table 10. To show the trace of spaces (width or length) in *CM_HM_*, we have highlighted them, but they are transparent in practice.

To evaluate the efficiency of the selected techniques, we implemented them on a simulated dataset. This dataset is generated by copying randomly some proverbs from referenced websites as outlined in Table 11 and Table 12. 

Let us assume that we wish to hide a *SM* = “original” or (64-bit) through the sample cover messages as depicted in Table 11. To evaluate the invisibility rate of selected algorithms, we analyzed them using equation (2) considering the differences between CM and CM_HM_ for each method that the obtained results listed in Table 13.

Since the majority of selected approaches embed the *SM_bits_* into the *CM* based on the bit-level marking (except MHST [29] & EM_ST [13]), we normalize the EC of each approach by considering 8-bit binary for each character of the *SM*. Moreover, we evaluate the embedding capacity of the selected algorithms based on the number of embeddable locations required to embed the *SM* in the *CM*.

**Figure 6:**
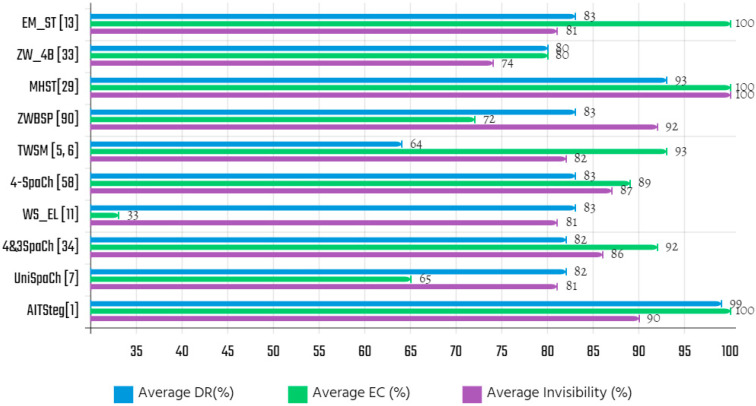
The overlap between the average Invisibility, EC and DR results (%).

Table 14 summarizes the EC rates offered by the evaluated approaches after analyzing them on the highlight samples (e.g., *SM* and *CM*). Assuming that a malicious user tampers with a word or a letter of the *CM_HM_*, then can the *SM_bits_* be extracted from the *CM’_HM_* by the extraction algorithm? To answer this question, we evaluated the approximate DR rate of each approach based on the embedding locations and the cover messages in Table 12 using equation (4) separately. The DR results listed in Table 15, and Figure 6 illustrates the average invisibility, EC and DR of evaluated techniques.

Table 16 depicts a comparative analysis of selected structural approaches in terms of criteria and language coverage along with their limitations. To demonstrate the efficiency of evaluated algorithms, we rated them according to the results concerning to invisibility, EC, and DR: for example, invisible, and visible for the invisibility; low, medium, and high scale for the EC; low, modest, and high for the DR.

In practice, all the approaches that work based on modifying the spaces between words, cannot be applied in Chinese texts because in this language there are no spaces between words.

To demonstrate the pros and cons, we considered four types of effective attacks for assessing their limitations such as visual (tampering), structural (formatting), statistical (decoding), and retyping attacks. Let us suppose that a malicious user copies a portion (or all) of the *CM_HM_* which included the *SM_bits_* into a new host text message/file and randomly modifies it in terms of mentioned attacks. In this case, if even one bit or character of the *SM* is altered, then it leads to the extraction of the *SM* by the corresponding *Ext()* to fail. Table 17 depicts the evaluated results conducted on the *CM_HM_* examples.

As shown in Table 17, almost all the evaluated algorithms have some limitations; however, some of them provide better safety than others. In practice, the programmers must take into account the priority of criteria in case of fragile or robust and, so, they choose a proper approach based on the security limitations which could give more safety in the particular application.

## 5. Suggestions for Future Works

Text hiding is a flexible and potent technique that could be employed in different ways to keep safe sensitive information in various areas such as covert communication, copyright protection, authentication, etc. Although the efficiency of text hiding algorithms has drawn much attention from cybersecurity researchers, it still lacks a precise analysis modeling which could take the fundamental criteria into account during the efficiency analysis.

As we already explained, there are four evaluation criteria for efficiency analysis, which rely on the way of embedding. In other words, the embedding methods generally specify how to evaluate the efficiency of the particular algorithm. Therefore, to assess the effectiveness of a specific algorithm, it is necessary to compare it with previous works within the same category (e.g., linguistic, structural, and random and statistics). We have also summarized the various limitations of three major types of text hiding techniques in Table 3, which provides a better understanding of the state-of-the-art and hopefully can guide in developing future works. Since many types of research concerning the structural-based techniques (only a few algorithms proposed in other categories) and affording better efficacy have been carried out, we have tried to highlight the recently proposed algorithms in this paper.

As we have pointed out in Section 3, the linguistic and random and statistics-based approaches have more limitations compared to structural-based methods. Due to the use of extra dictionaries and high computational complexity, a few researchers focused on linguistic and random and statistics-based approaches in recent years as well. Over the last decade, many structural-based algorithms have been introduced to improve the efficiency of text hiding by considering the optimum trade-off between criteria, as depicted in Table 16 and Table 17. However, the embedding capacity and robustness of them require to be more improved against various attacks regarding security requirements. In what follows, we recommend some guidelines aimed at instructing cybersecurity researchers on the best options to apply the structural based algorithms relying on the characteristics of the applications. Nevertheless, we have to declare that these recommendations are general and empirically derived rules of thumb; these directions should not be considered rigidly or dogmatically.

- Since most of the authentication systems utilize SMS to verify the authenticity of users, the structural-based technique can be employed as the best option to provide covert communication against unpredictable network attacks such as MITM, brute-force, and guessing attacks.
- Where the primary concern is the invisible transmission of secret information over public networks, the structural-based steganography algorithms could be utilized for providing that requirement.
- In the case of unauthorized access tracking, a combination of machine learning algorithms and the ZWC-based methods can be employed to mark sensitive documents over private networks. For instance, confidential documents in a governmental organization could be marked with identifiers such as an invisible signature which is difficult to detect.
- Due to the fact social media have become a significant part of the end users’ daily communications, a combination of unsupervised learning algorithms and structural-based text hiding can be used to intelligent information analysis during the resharing/reproduction of data to protect valuable information against malicious attacks.
- The lossless compression algorithms such as Huffman coding, LZW, arithmetic, and so on, could be utilized during the encoding section of structural-based methods to improve the embedding capacity criteria. An efficient text hiding algorithm should provide optimum trade-off among the three fundamental criteria to gain a certain level of security.
- To sum up, which type of text hiding algorithms provides better efficiency? We cannot give an accurate and unique answer to this question. Cybersecurity researchers must take into account many things like various pros and cons of text hiding algorithms, together with the recommendations that we have outlined. Also, they should ponder whether the text hiding techniques would be relevant or not for the particular application. When the researcher comprehends that some of the merits of a specific algorithm could provide a proper benefit to the exact needs of the application at issue; hence it should probably be given a try.

## 6. Conclusions

This case study presents a comparative analysis of existing text hiding techniques, especially on those focused on modifying the structural characteristics of digital text message/file. We overviewed a range of fundamental criteria, applications, and attacks covering the text hiding area to explain the current security challenges in the cybersecurity industry. Also, we summarized three major categories of text hiding techniques based on how to process cover text messages/files to embed the secret bits, namely, structural, linguistic, and random and statistics. We then outlined the limitations and characteristics of each category to show their efficiency in various applications. Moreover, we evaluated the recently proposed approaches concerning the fundamental criteria to highlight their pros and cons. Finally, we have recommended some of guidelines and directions that merit further attention in future works.

## Figures and Tables

**Table 1:** The most utilized special Unicode characters in recent introduced techniques.

Algorithm	Name	Hex Code	Decimal Code	Written Symbol
[1,27,28,33,42,55,58,91]	Zero-Width-Non-Joiner	U+200C	8204	No symbol and width
[1,4]	POP Directional	U+202C	8236	No symbol and width
[1,4]	Left-To-Right Override	U+202D	8237	No symbol and width
[1,28,33,42]	Left-To-Right Mark	U+200E	8206	No symbol and width
[4]	Right -To- Left Override	U+202E	8238	No symbol and width
[5,6,53,54,91]	Narrow No-Break Space	U+202F	8239	No symbol and width
[55,56]	Left-to-right embedding	U+202A	8234	No symbol and width
[55,56]	Right-to-left embedding	U+202B	8235	No symbol and width
[7,55,56]	Mongolian-vowel separator	U+180E	6158	No symbol and width
[28,33]	Right -To- Left Mark	U+200F	8207	No symbol and width
[28,33,42,55,56]	Zero-Width-Joiner	U+200D	8205	No symbol and width
[42,55,56,58]	Zero-Width-Space	U+200B	8203	No symbol and width
[55,56]	Zero-Width-Non-Break	U+FEFF	65279	No symbol and width
[5,6,7,27,34,53,54,58]	Hair Space	U+200A	8202	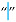
[5,6,7,27,34,54]	Six-Per-Em Space	U+2006	8198	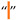
[5,6,7,27,34,54]	Figure Space	U+2007	8199	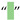
[5,6,7,27,34,54]	Punctuation Space	U+2008	8200	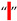
[5,6,7,34,54,58]	Thin Space	U+2009	8201	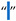
[5,6,7,34,54]	En Quad	U+2000	8192	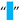
[5,6,7,34,54]	Three-Per-Em Space	U+2004	8196	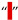
[5,6,7,34,54]	Four-Per-Em Space	U+2005	8197	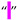
[5,6,7,27,34,100]	Normal Space	U+0020	32	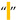

**Table 2:** Text Character Limitation of Social Media and Messenger apps [1,6].

Number	Social Media or Messenger Name	Message/Post	Text Limits Number of ASCII Characters	Text Limits Number of UTF-8 Characters
1	SMS	Message	2048	1024
2	Facebook	Wall Post	63,206	31,603
3	LinkedIn	Post	52,286	29,718
4	Twitter	Tweet	280	140 (Exclusive encoding)
5	Google+	Post	100,000	50,000
6	Instagram	Pic Caption	2200	1100
7	Pinterest	Pin Description	500	250
8	YouTube	Video Description	5000	2500
9	WhatsApp	Message	30,000	30,000
10	Gmail	Mail Text	35,000,000	35,000,000
11	WeChat	Message	16,207	16,207
12	Imo	Message	Virtually Unlimited	Virtually Unlimited
13	Hangouts	Message	Virtually Unlimited	Virtually Unlimited
14	Telegram	Message	4096 (Exclusive encoding)	4096 (Exclusive encoding)
15	Line	Message	10,000	10,000
16	Tango	Message	520	520
17	QQ	Message	16,207	16,207

**Table 3:** Highlighted pros & cons of various types of text hiding techniques concerning criteria.

Type Name	Invisibility	EC	DR	Language Coverage	Pros & Cons
Linguistic [17,62,70,71,80,81,82,83,84,85,106,109]	Imperceptible	Low	Medium	Exclusive	➢ Having high complexity due to using an additional dictionary to replace the words/characters in the CM. ➢ Altering the meaning of original CM after embedding an SM. ➢ Depending on an exclusive language (e.g., English, Persian/Arabic, etc.) ➢ Providing high invisibility, Low EC (e.g., 1 bit per synonym), and Medium robustness against visual attacks.
Structural [1,2,3,4,5,6,7,8,11,20,34,41,54,65,66,100,112,113,114]	Imperceptible	High	High	Multilingual	➢ Having no perceptible changes on the original CM after embedding an SM. ➢ Increasing the length of the CM by embedding additional Unicode invisible symbols. ➢ Depending on the encoding features of the CM (e.g., not the CM content, or language). ➢ Providing high invisibility (except color based methods), higher EC (e.g., n-bit per location), and high robustness against structural and visual attacks.
Random & Statistics [21,23,24,29,34,35,39,47,51,124]	Perceptible	Modest	High	Exclusive	➢ Having high complexity due to employing an extra compression algorithm to encode the *SM_bits_*. ➢ High robustness against visual attacks ➢ Depending on the language of the *CM.* ➢ Providing perceptible transparency (low invisibility), modest EC, and high robustness against visual attacks

**Table 4:** Highlights & Limitations of various types of text hiding techniques.

Type	Hidden Transmission	Network Cover Channels	Unauthorized Access Detection	Highlights and Limitations
Linguistic	✓	✓	×	➢ The linguistic-based methods are not applicable to unauthorized access detection due to altering the original meaning of the CM during the embedding an SM. ➢ For employing in covert channels, they need a long CM, and can only be used in a CM with exclusive language. ➢ For utilizing in hidden transmission, they are not enforceable in limited communication channels.
Structural	✓	✓	✓	➢ The structural-based approaches can provide all of three applications. ➢ For utilizing in hidden transmission, they are not applicable in limited communication channels. ➢ Due to employing language-independent features of the *CM* to embed the SM, these methods could be used in multilingual texts.
Random & Statistics	✓	✓	×	➢ The random cover--based algorithms are not applicable to unauthorized access detection due to generating an unknown CM. ➢ For applying in hidden transmission, the generated CM raises suspicions for attackers. ➢ Due to generating a CM based on the SM, these approaches could only be applied to secure an SM with exclusive language.

**Table 5:** Sample of Hidden Bits by using Word Symbols in [33].

Right to Left Mark	Left to Right Mark	ZWJ	ZWNJ	SM_bits_
×	×	×	×	0000
×	×	×	-	0001
×	×	-	×	0010
×	×	-	-	0011
…	…	..	..	…

**Table 6:** Predefined pattern of embedding location in [90].

2-Bit	Embeddable Location
‘00′	No ‘ZWC’ + ”U+0020”
‘01′	“U+0020” + No ‘ZWC’
‘10′	“U+200B” + ”U+0020”
‘11′	“U+0020” + “U+200B”

**Table 7:** Unicode ZWCs 2-bit classification pattern in [1].

2-Bit Classification	Hex Code
00	U+200C
01	U+202C
10	U+202D
11	U+200E

**Table 8:** Mapping Pattern of SMbits for marking the inter-word and inter-sentence locations in [34].

Spaces Pattern	4-bit Classification
Normal Space	0000
Normal Space + Three-Per-Em	0001
Three-Per-Em + Normal Space	0010
Normal Space + Four-Per-Em	0011
Four-Per-Em + Normal Space	0100
Normal Space + Six-Per-Em	0101
Six-Per-Em + Normal Space	0110
Normal Space + Figure	0111
Figure + Normal Space	1000
Normal Space + Thin	1001
Thin + Normal Space	1010
Normal Space + Hair	1011
Hair + Normal Space	1100
Normal Space + Punctuation	1101
Punctuation + Normal Space	1110
Normal Space + Narrow No-Break	1111
Narrow No-Break + Normal Space	1111

**Table 9:** Mapping Pattern of *SM_bits_* for marking the inter-paragraph and end of line locations in [34].

Spaces Pattern	3-bit Classification
Three-Per-Em Space	000
Four-Per-Em Space	001
Six-Per-Em Space	010
Figure Space	011
Punctuation Space	100
Thin Space	101
Hair Space	110
Narrow No-Break Space	111

**Table 10:** Implementation of selected structural approaches on the highlight examples.

Algorithm	CM	CM_HM_	Embedded SM_bits_
AITSteg [1]	The only source of knowledge is experience.	The only source of knowledge is experience.	12
ZW_4B [33]	The only source of knowledge is experience.	The only source of knowledge is experience.	16
MHST [29]	The only source of knowledge is experience.	The only source of knowledge is experience.	0
ZWBSP [90]	The only source of knowledge is experience.	The only source of knowledge is experience.	12
TWSM [5,6]	The only source of knowledge is experience.	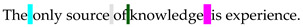	16
4-SpaCh [58]	The only source of knowledge is experience.	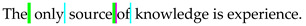	16
WS_EL [11]	The only source of knowledge is experience.	The only source of knowledge is experience.	6
4&3SpaCh [34]	The only source of knowledge is experience.	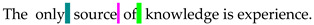	16
UniSpaCh [7]	The only source of knowledge is experience.	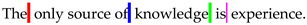	16
EM_ST [13]	The only source of knowledge is experience.	The 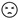 only 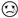 source of knowledge is experience.	16

**Table 11:** Dataset: cover message examples.

Name	Text Content	Reference
CM.1	Science without religion is lame, religion without science is blind.	https://www.brainyquote.com
CM.2	君子之行，静以修身，俭以养德，非澹泊无以明志，非宁静无以致远。《诫子书》	https://www.fluentu.com/
CM.3	Die größte Gefahr für die meisten von uns ist nicht, dass wir hohe Ziele anstreben und sie verfehlen, sondern dass wir uns zu niedrige setzen und sie erreichen.	https://www.germanpod101.com
CM.4	جهان سوم جایی است که هر کس بخواهد مملکتش را آباد کند، خانه اش خراب می شود و هر کس بخواهد خانه اش را آباد کند باید در ویرانی مملکتش بکوشد.	http://www.bartarinha.ir/
CM.5	Chi vuol andar salvo per lo mondo, bisogna aver occhio di falcone, orecchio d’asino, viso di scimia, bocca di porcello, spalle di camello, è gambe di cervo.	http://oaks.nvg.org/

**Table 12:** The detailed structures of sample cover texts.

Cover Name	Characters	Spaces	Words	Sentences	Lines	Language
CM.1	68	9	10	1	2	English
CM.2	36	0	36	1	2	Chinese
CM.3	160	27	28	1	4	German
CM.4	137	30	31	1	3	Persian
CM.5	156	26	27	1	4	Italian

**Table 13:** Invisibility (%) Analysis of evaluated methods using Jaro Distance based on the examples.

Algorithm	CM.1	CM.2	CM.3	CM.4	CM.5	Average Invisibility (%)
AITSteg [1]	89.3	84.3	94.4	89.3	95.1	≅90
UniSpaCh [7]	83.8	0	80.8	79.9	80.4	≅81
ZW_4B [33]	62.5	47.2	94.0	0	93.4	≅74
MHST [29]	100	0	100	100	100	≅100
ZWBSP [90]	96.1	0	95.1	80.1	95	≅92
TWSM [5,6]	85.7	0	81.8	79.3	80.7	≅82
4-SpaCh [58]	82.9	0	84	84.1	96.5	≅87
WS_EL [11]	83.4	0	81.1	80.3	80.6	≅81
4&3SpaCh [34]	84.9	0	87	87.5	84.6	≅86
EM_ST [13]	83.2	0	81.1	80.1	80.1	≅81

**Table 14:** EC (Bit & %) results of structural approaches on the highlight samples.

Algorithm	Type of Embedding	CM.1	CM.2	CM.3	CM.4	CM.5	Average EC/64 (%)
AITSteg [1]	Bit-level	64	64	64	64	64	≅ 64 => 100
UniSpaCh [7]	Bit-level	22	4	62	64	60	≅ 42 => 65
ZW_4B [33]	Bit-level	64	64	64	0	64	≅ 51 => 80
MHST [29]	Character-Level	8*8 = 64	0	8*8 = 64	0	8*8 = 64	≅ 64 => 100
ZWBSP [90]	Bit-level	18	0	56	60	52	≅ 46 => 72
TWSM [5,6]	Bit-level	47	0	64	64	64	≅ 60 => 93
4-SpaCh [58]	Bit-level	36	0	64	64	64	≅ 57 => 89
WS_EL [11]	Bit-level	11	2	31	33	31	≅ 22 => 33
4&3SpaCh [34]	Bit-level	45	9	64	64	64	≅ 59 => 92
EM_ST [13]	Character-Level	8*8 = 64	0	8*8 = 64	8*8 = 64	8*8 = 64	≅ 64 => 100

**Table 15:** Approximate DR (%) results of evaluated approaches on the highlight samples.

Algorithm	CM.1	CM.2	CM.3	CM.4	CM.5	Average DR (%)
AITSteg [1]	98.5	97.2	99.3	99.2	99.3	≅99
UniSpaCh [7]	83.8	88.8	80.6	75.9	80.7	≅82
ZW_4B [33]	76.4	55.5	90	88.3	89.7	≅80
MHST [29]	88.2	0	95	0	94.8	≅93
ZWBSP [90]	86.7	0	83.1	78.1	83.3	≅83
TWSM [5,6]	57.3	0	66.8	78.1	51.9	≅64
4-SpaCh [58]	86.7	0	83.1	78.1	83.3	≅83
WS_EL [11]	83.8	95	80.6	75.9	80.1	≅83
4&3SpaCh [34]	82.3	91.6	80	75.1	80.1	≅82
EM_ST [13]	86.7	0	83.1	78.1	83.3	≅83

**Table 16:** Comparative analysis of structural approaches in terms of criteria and language coverage.

Algorithm	EC	DR	Invisibility	Limitations	Language Coverage
AITSteg [1]	High	High	Imperceptible	Embeds additional ZWCs in front of the CM	Multilingual
UniSpaCh [7]	Low	Medium	Imperceptible	Depends on the spaces between words	Multilingual
ZW_4B [33]	Modest	Medium	Imperceptible	Embeds four ZWCs after each letter	Exclusive (Latin)
MHST [29]	High	High	Imperceptible	Depends on using an exclusive language in the *SM*	Exclusive (Latin)
ZWBSP [90]	Low	Medium	Imperceptible	Depends on the spaces between words	Multilingual
TWSM [5,6]	High	Low	Imperceptible	Depends on the spaces and font style of the *CM*	Exclusive (Latin)
4-SpaCh [58]	Modest	Medium	Imperceptible	Depends on the spaces between words	Multilingual
WS_EL [11]	Low	Medium	Imperceptible	Embeds two spaces between words	Multilingual
4&3SpaCh [34]	High	Medium	Imperceptible	Depends on the spaces between words	Multilingual
EM_ST [13]	High	Medium	Visible	Embeds additional emoticons between words	Multilingual

**Table 17:** A comparison analysis of evaluated techniques against the stated attacks.

Algorithm	Having Robustness Against Attack: Yes (✓) and No (×)				Security Limitations
	Visual	Structural	Statistical	Retyping	
AITSteg [1]	✓	✓	✓	×	Optimum safety (3)
UniSpaCh [7]	✓	✓	✓	×	Optimum safety (3)
ZW_4B [33]	×	✓	✓	×	Medium safety (2)
MHST [29]	×	✓	✓	×	Medium safety (2)
ZWBSP [90]	✓	✓	✓	×	Optimum safety (3)
TWSM [5,6]	×	×	✓	×	Easy to lose (1)
4-SpaCh [58]	✓	✓	✓	×	Optimum safety (3)
WS_EL [11]	✓	✓	✓	×	Optimum safety (3)
4&3SpaCh [34]	✓	✓	✓	×	Optimum safety (3)
EM_ST [13]	×	✓	✓	×	Medium safety (2)
